# Understanding the Relative Impact of Dual Identification on Brand Loyalty on Social Media: The Regulatory Fit Perspective in Different Cultures

**DOI:** 10.3389/fpsyg.2022.901706

**Published:** 2022-06-14

**Authors:** Shang Chen, Qingfei Min, Xuefei Xu

**Affiliations:** ^1^School of Economics and Management, Dalian University of Technology, Dalian, China; ^2^Faculty of Humanities and Social Sciences, Dalian University of Technology, Dalian, China

**Keywords:** brand loyalty, dual identification, regulatory focus, culture, IT affordance, social media

## Abstract

This study explorers whether the relative impacts of brand identification and identification with other users of brand pages on brand loyalty vary according to consumers’ regulatory focus. By integrating social identification theory with regulatory focus theory, this study adopts a dual identification framework to compare the differential impacts of promotion regulatory fit and prevention regulatory fit on brand loyalty. Besides, the moderating effects of product type on the relationship between promotion/prevention regulatory fit and brand loyalty are further investigated. Finally, this study uses different combinations of information technology (IT) affordances in order to examine their influences on each identification target. The current study adopts a qualitative methodology and involved conducting semi-structured interviews with 27 brand page users in regard to IT affordances and their subdimensions. The research model was empirically tested using a cross-country comparison of data collected from surveys conducted in China and the United States. The results support our hypotheses and confirm the differential effects of promotion and prevention regulatory fit on brand loyalty. Theoretically, our study enhances our understanding of the relative effect of dual identification on brand loyalty on social media. Practically, our study delivers insights for companies into how social media brand pages can be used as a strategic tool to achieve brand values.

## Introduction

In the fierce competitive environment, social media platforms have been actively used by companies to present their brand online and achieve certain marketing values (Santos et al., 2022). Social networking sites are a popular form of social media platform that enable communication with others (Nadeem et al., 2021). The rapid growth of social networking sites such as Facebook and WeChat has attracted the attention of companies, who use the platforms to create brand pages (SBP). A brand page (also known as a fan page) is essentially a profile created and operated by a company on a social networking site to promote corporate brands and engage with existing and potential customers. Thus, many prior studies have investigated the independent impacts of either brand identification or identification with other SBP users on brand loyalty within SBPs (Kyu et al., 2018; Warner-Soderholm et al., 2018; Martínez-López et al., 2021). However, few studies have compared the effects of two identification targets in an integrative framework; the literature thus fails to identify specific characteristics related to consumers’ identification in the context of SBPs.

In practice, a brand itself and other users of SBPs that are essentially different in nature can coexist in regard to SBPs and thus need to be managed holistically. On the one hand, brands tend to highlight product benefits and downplay weaknesses to create a favorable brand reputation and image. Prior studies show that an attractive brand image can satisfy the needs of consumers for self-reinforcement, thereby increasing brand loyalty (Zhang, 2021). On the other hand, consumers also have the opportunity to communicate and socialize with other users of the SBPs, which is more likely to create a group identity than a unique identity related to the self (Guegan et al., 2017). Since consumers see themselves as being members of specific social groups, they possess duties, social norms, or responsibilities set by those groups (Huang et al., 2019). Thus, these two types of identification are seen as unique entities by users of SBPs. As these different forms of identification interact and could conflict with each other, there is a gap in our understanding regarding their differential roles in the context of SBPs. To address this concern, we use a dual identification model that encompasses identification with brands, as well as other users of SBPs, in order to empirically examine the relative efficacy of the two identification targets in terms of strengthening brand loyalty.

More importantly, the relative salience (efficacy) of each target of identification is fluid and depends largely on consumers’ personalities, such as their regulatory focus (Jones and McEwen, 2000). Regulatory focus is a personal trait. It suggests that human behavior revolves around two approaches: promotion focus concerns gains, aspirations, and achievements, and prevention focus concerns losses, responsibilities, and safety (Higgins, 1997). Two people who have different forms of regulatory focus will experience different things when facing multiple forms of identification. If a certain target of identification causes individuals to experience regulatory fit, positive feelings are elicited, which enhances subsequent brand loyalty (Cesario et al., 2004). Despite logical links between separate identification targets, regulatory focus, and brand loyalty, there is scarce empirical research exploring these relationships in the context of SBPs. Given that promotion is emphasized in Western cultures and prevention is emphasized in Eastern cultures (Thongpapanl et al., 2018), we address this gap by investigating the effects of regulatory fit on the relative importance of two identification targets in both the United States and China. As such, the first research question of this article is as follows.

RQ1: Do the relative impacts of the two identification targets on brand loyalty depend on regulatory fit?

Additionally, previous studies show that contextual factors can affect regulatory fit (Bullard and Penner, 2017). In recent years, as more and more companies have adopted SBPs to manage and market their various types of products, the regulatory fit effects described above may be affected by the brand’s product type. Peterson et al. (1997) categorized online products into two types: search products and experience products. When the product type of a brand matches an individual’s regulatory focus, that individual will find it easier to positively evaluate the brand (Hua et al., 2017). Specifically, promotion-focused individuals tend to prefer experience products, whereas prevention-focused individuals are more likely to select search products (Alexander, 2004; Hua et al., 2017). For this reason, our study further investigates whether and how product type moderates the relationship between consumers’ regulatory fit with different targets of identification and brand loyalty. As such, our second research question is as follows.

RQ2: How does product type moderate the relationship between promotion/prevention regulatory fit and brand loyalty?

Finally, because of the differences in nature between two identification targets, questions of how the antecedents of brand identification differ from those of identification with SBP users are equally as intriguing. Social identity theory suggests that identification is formed “through a complex interplay of cognitive, affective, and social interaction processes, occurring within particular local contexts” (Vignoles et al., 2006). Identification is in tune with the affordance of the context at hand, with an affordance being the “multifaceted relational structure” between an object or piece of technology and the user; this structure facilitates or restricts behavioral outcomes that context (Piccoli, 2016). This study therefore uses the perspective of affordance to examine the antecedents of two identification targets. More importantly, we qualitatively analyze in-depth interviews with SBP users to explore their affordances. As such, our third research question is as follows.

RQ3: How do different combinations of IT affordances influence each target of identification?

Our research contributes to existing literature in the following ways. First, this study is distinguished from previous studies that have generally only focused on either brand identification or identification with SBP users. We simultaneously consider these two different targets of identification in order to gain a thorough understanding of their relative importance in explaining brand loyalty. Second, this study develops regulatory focus theory by investigating the effects of the fit between regulatory focus and two identification targets on brand loyalty. More importantly, as cultural background can foster distinct regulatory orientations, we move away from the single-country perspective and empirically examine the role of customers’ regulatory fit in an SBP context across Western and Eastern cultures. Third, this study also empirically tests how two different brand-related product types moderate the relationship between regulatory fit and brand loyalty. This expands previous studies, which take it for granted that different brands have the same attributes (Wang et al., 2018). Finally, this research develops our understanding of IT affordances. Little research has explored how and why IT affordances are related to building social identification. We therefore analyze the influence of IT affordances on two targets of identification in the context of SBPs.

This article is structured as follows. First, the theoretical foundation used to frame this research is introduced. Then, a research model is proposed, and detailed hypotheses are presented. Subsequently, the research methodology is described. The results of data analysis are then reported, followed by a discussion of the results. The paper ends with a conclusion regarding the study’s theoretical and managerial implications, its possible limitations, and future research.

## Theoretical Development

### Dual Identification and Brand Loyalty

Social identification concerns a consumer’s psychological state of perception, feelings, and values in regard to his or her belonging to a group or entity (Hsu et al., 2015). Although recent studies have demonstrated that a consumer’s identification with brands or SBP users can act as an important driver of brand loyalty (Hua et al., 2017; Kyu et al., 2018; Warner-Soderholm et al., 2018), these studies mostly focus on only a single aspect of consumers’ identification, rather than multiple aspects. Social identity theory implies that people have multiple social identities and are therefore likely to categorize themselves into different social groups (Higgins, 1997). Hence, one may simultaneously identify with multiple targets of identification. Based on this, Río et al. (2001) further explain multiple aspects of identification via a dual identification framework, which includes group identification and individual identification. In the present research, two social references (the brand itself and other users of the SBPS) coexist within SBPs. Therefore, social categorization processes might lead to individuals’ identification with a brand and other SBP users, respectively.

However, these multiple identifications exist in a hierarchy in which prevalent forms of identification have greater influence over individual behavior in a specific situation (Arnett et al., 2003). That is, the relative impacts (salience) of each identification target are fluid and change depending on the different dimensions of brand loyalty in terms of repurchases and recommendations for the brand within SBPs. While these two dimensions of loyalty collectively contribute to the building of a successful brand, they essentially belong to different role behaviors (Levy et al., 2021). Role expectation has been used to identify two types of consumers’ role behaviors: in-role behavior, which refers to the formal part of a consumer’s activity, and extra-role behavior, which refers to voluntary and unrewarded behavior (Chen et al., 2010). In this study, the repurchasing of products, analogous to in-role behavior, is expected and required for consumers to satisfy their basic psychological needs, and is associated with customer role. Conversely, similar to extra-role conduct, brand recommendation extends beyond role expectations and responsibilities, and is involved in activities that are not formally required. In other words, the two dimensions of brand loyalty bring different perceived value to consumers (Hopkins, 2022; Kliestik et al., 2022b). Therefore, although the two targets of identification can enhance different dimensions of brand loyalty, certain identification targets may have a greater impact on repurchasing products, whereas others may be more influential in terms of brand recommendation (Lazaroiu et al., 2020; Nica et al., 2022). We explain the differential effects of the two identification targets on in-role and extra-role loyalty behaviors in more detail in the hypotheses section.

### Achieving Regulatory Fit With Two Identification Targets

Higgins (1997) proposed regulatory focus theory to posit that the goal-oriented behavioral patterns of individuals are impacted by two different motivational systems consisting of a promotion-focus orientation and a prevention-focus orientation. Promotion focus occurs as a result of needs related to nurturance, such as growth and attainment. The responsive behaviors involved in promotion-focus motivations are related to the approach orientation. In contrast, prevention focus occurs as a result of needs related to security, such as the aversion to loss and risks. The responsive behaviors involved in prevention-focused motivations concern the avoidance orientation. That is, when individuals are motivated by promotion goals, they strive toward being their “ideal self.” This state is related to their hopes and desires. It impacts the way they behave and drives them to pursue positive goals with the aim of self-enhancement (Hua et al., 2017). Alternatively, individuals driven by prevention goals tend to adapt their behaviors in order to avoid negative outcomes. This process enables them to strive toward their “ought self,” which is related to their responsibilities and obligations (Craciun, 2018). In a nutshell, since promotion-focused and prevention-focused individuals tend to differ in terms of their psychological states and cognitive processes (Kliestik et al., 2022a; Rowland, 2022), regulatory focus can influence their decision-making. As discussed in the previous section, repurchase intention and brand recommendations have distinct perceived risks and benefits, as they essentially belong to different role behaviors. According to regulatory focus theory, if a certain dimension of loyalty matches an individual’s regulatory focus, they will experience regulatory fit. Individuals with higher levels of regulatory fit gain more value from fit and are more likely to put extra effort into pursuing their goals (Zvarikova et al., 2022). According, the relative impact of the two identification targets on each dimension of brand loyalty depends on consumers’ different regulatory focuses.

On the one hand, higher levels of brand identification mean that a brand has a more prestigious identity (Peng et al., 2014). A favorable identity can fulfill the self-enhancement, self-distinctiveness, and self-esteem of promotion-focused individuals, because they are attuned to positive results (He et al., 2012a). That is, brand identification matches individuals’ goal pursuit orientations in terms of their achievements and aspirations. Thus, promotion-focused customers are more willing to repurchase a brand to express their ideal self-identity to others when they identify with a specific brand. Simultaneously, the more prestigious a brand’s identity is, the more it can help prevention-focused individuals reduce uncertainty about a brand’s products and services. This can greatly reduce the risk perceived by prevention-focused individuals when they recommend an identified brand. In this context, regulatory fit occurs and prevention-focused individuals have a “good feeling,” thereby significantly enhancing their recommendation intention.

On the other hand, identification with SBP users creates a sense of identification with a social group, enabling group members to perceive their shared identity. As a result, identification with SBP users concerns psychological status, which views users as a collective, as opposed to individuals. Recommending brands in a group can provide useful information to others, which can enable promotion-focused individuals to gain status and reputation. Thus, identification with SBP users is more likely to motivate them to consistently recommend brands due to the regulatory fit effect. Alternatively, identification with SBP users also signifies individuals’ assimilation with a specific group, by which the thoughts and behaviors of the group become the individuals’ too (Mikal et al., 2014). Once individuals identify with SBP users, they are more likely to comply with the group’s rules and obligations, to ensure psychological safety (Farivar et al., 2018). In other words, identification with SBP users can reduce prevention-focused individuals’ uncertainty in regard to purchasing products. This should create regulatory fit and increase the possibility of product repurchase among prevention-focused individuals.

More crucially, as more and more SBPs are engaging with consumers, the product categories of different brands are different. According to the product features, products can be classified into two categories: search versus experience products (Phillip, 1970). With search products, consumers can acquire complete information about a product prior to purchasing it, whereas, with experience products, it is not possible for consumers to acquire information about product attributes until they have acquired and used one of the products.

According to the definitions of search and experience products, risk difference constitutes a significant discrepancy between the two types of products. As individuals with different regulatory focuses have different attitudes toward perceived risks, the effects of both promotion and prevention regulatory fit on brand loyalty depend largely on a brand’s product type (Hsu et al., 2017). Thus, the present study further explores whether the regulatory fit effects described above vary across conditions. Although products can be divided into other categories, such as convenience products vs. specialty products or hedonic products vs. functional products, these product categories fail to distinguish differences in consumers’ perceptions of product risk. Thus, this study only considers the classification of search products and experience products, without considering other product classifications.

### Dual Identification and IT Affordance

Social identity theory implies that individuals possess more than one dimension of identity, and all of these dimensions are socially constructed (Tajfel and Turner, 1986). Identity also exists along a continuum, with interpersonal behavior at one end (personal identity) and intergroup behavior at the other (social identity). Thus, an individual’s identity is influenced by the changing contexts around them, which also impact the prominence of multiple dimensions of identity. Because similarity breeds attraction, people are more willing to identify with targets that match their identity. That is, social identification is not static and cannot be treated as such; where technological elements may remain the same, users’ perceptions of those elements will change over time (Leonardi, 2011). IT affordances, constituting the connections between users’ perceptions and technological capabilities (Leonardi, 2011; Majchrzak et al., 2013), are useful in an explanation of how IT features foster social identification within SBPs. We therefore employed semi-structured interviews with SBP users under a qualitative methodology in order to identify affordances related to two identification targets. Specifically, the outcomes of the interviews show that “metavoicing” and “social connecting” are associated with identification with SBP users, but “visibility,” “metavoicing,” and “triggered attending” are associated with brand identification. We discuss these relationships in detail in the hypotheses section.

## Theoretical Model and Hypotheses Development

### IT Affordances and the Two Identification Targets

The affordance of visibility suggests that SBP can effectively provide relevant or useful information to users (Zheng et al., 2013). This information ensures that product pictures and information are accessible to users, mitigating the risks of product uncertainty, which in turn reduces divergence between customers and brands. The visibility affordance can also make the presentation of the brand itself more vivid, which is useful for attracting the attention of consumers. Users will therefore tend to identify with a brand as a result of following the brand’s SBP. Thus, we hypothesize the following.

**Hypothesis 1:** *The visibility affordance has a positive influence on identification with a brand.*

The affordance of metavoicing ensures that users and brands interact, as well as providing feedback on products (Ou et al., 2014). Through the metavoicing affordance, both users and brands are able to listen to and negotiate with each other, which in turn enables both parties to attain a satisfactory mutual understanding. Thus, metavoicing has the potential to be an important influence on building brand identification. Additionally, the metavoicing affordance increases individuals’ retweeting behavior, which offers other users the opportunity to engage productively in an ongoing conversation (Kim et al., 2014). In other words, metavoicing is vital in enhancing communication between users and brands. In the process, it enables identification with SBP users. We therefore hypothesize the following.

**Hypothesis 2a:** *The metavoicing affordance has a positive impact on identification with brands.*

**Hypothesis 2b:** *The metavoicing affordance has a positive impact on identification with SBP users.*

The affordance of triggered attending informs users about changing content concerning products and services (Alba et al., 1997). It can improve brands’ ability to interact with users at crucial times during service encounters. This information can prompt users to become curious about the changes; thus, users are likely to engage more with brands in order to acquire up-to-date information. In other words, the triggered affordance creates opportunities for conversations between users and brands, contributing to brand identification. Thus, we hypothesize the following.

**Hypothesis 3:** *The triggered-attending affordance has a positive impact on identification with brands.*

The affordance of social connection creates new connections between users (Chiu et al., 2006). Once the connection occurs, the SBP users can communicate with each other. That is, a high level of connectedness leads to users transforming temporary relationships into long-term connections. Social connectedness therefore constitutes a sense of belonging, as well as “the subjective awareness of being in close relationships with others” (Grieve and Kemp, 2015). It involves a form of social capital revolving around bonding, which in turn enables identification with other SBP users. We therefore hypothesize the following.

**Hypothesis 4:** *The social connection affordance has a positive impact on users’ identification with other SBP users.*

### The Effects of Regulatory Fit on Brand Repurchasing

#### The Effects of Promotion Regulatory Fit on Brand Repurchasing

Promotion-focused individuals strive toward personal ideals and are in tune with positive outcomes (Higgins, 1997). Within SBPs, individuals can simultaneously obtain marketer-generated content (MGC) from the brand and user-generated content (UGC) from other SBP users in order to learn about related products and services (Goh and Heng, 2013). Thus, the purchase decisions of individuals are jointly influenced by both MGC and UGC because they typically encounter information from multiple sources. Specifically, MGC tends to highlight the positivity of products, while downplaying weaknesses, given that there are underlying commercial motivations (Wu et al., 2009). For promotion-focused individuals, MGC that emphasizes product benefits is more effective. Brand identification leads to users becoming attached to a brand, which encourages them to devote substantial attention to MGC (Lam et al., 2010). Thus, brand identification is likely to impact promotion-focused consumers’ aim of achieving certain benefits (Prooijen et al., 2021).

Brand identification also means that the prestige of brand identity is perceived by consumers. A favorable brand identity can help promotion-focused consumers to shape their social identities. They can attain a more positive self-image and, in turn, obtain trust and respect (King et al., 2016). Individuals thus fulfill their needs related to self-esteem, because their relationship with the brand enables them to create their ideal self-image (He et al., 2012a). Based on the above discussion, brand identification (vs. identification with SBP users) should create promotion regulatory fit and lead to greater repeat purchase intention among promotion-focused consumers. Accordingly, the following hypothesis is proposed.

**Hypothesis 5a:** *Identification with a brand has more of an impact on promotion-focused individuals than it does on SBP users.*

#### The Effects of Prevention Regulatory Fit on Brand Repurchasing

Since prevention-focused individuals are less tolerant of risk, they have a greater tendency to take others’ opinions on board to navigate any uncertainty when shopping (Higgins, 1997). In contrast to MGC, UGC regarding SBPs is provided by any users who have an interest in interacting with the businesses at hand, or other customers. UGC is thus typically formed of open expressions, in that users can discuss a company’s products and complain about them (Yang et al., 2019). For prevention-focused consumers, messages that focus on loss avoidance are thus more relevant, as they enable them to limit uncertainty when making decisions about purchases. Identification with users of SBPs creates a sense of belonging, which prompts individuals to engage in positive and cooperative ways with other group members (Tsai and Hung, 2019). This enables prevention-focused individuals to gain greater access to UGC regarding SBPs, thereby increasing the possibility of repurchasing.

Additionally, due to their strong focus on loss prevention and vigilance strategies, prevention-focused individuals pay more attention to duties, social norms, and responsibilities that are generally interpersonal (Tsai and Hung, 2019). Thus, they tend to conform to the social consensus at hand, believing that it enables them to avoid criticism from others. Identification with SBP users means that the consumer agrees with the group’s norms, traditions, rituals, and objectives (Zhang et al., 2021), which makes them conform to what others around them do or do not believe. In summary, identification with SBP users (vs. brand identification) should create prevention regulatory fit and lead to greater repeat purchase intention among prevention-focused consumers.

**Hypothesis 5b:** *Identification with SBP users has more of an effect on repurchase intention for prevention-focused individuals than brand identification does.*

### The Effects of Regulatory Fit on Brand Recommendation

#### The Effect of Promotion Regulatory Fit on Brand Recommendations

Identification with SBP users creates a sense of belonging with the group; in-group members thus feel an attachment to each other and understand their mutual interests (Ho, 2015). That is, in-group membership establishes a common identity and thus enables in-group connections (Trepte, 2006). Hence, compared with brand identification, identification with SBP users is more likely to increase promotion-focused individuals’ awareness of the possible gains of recommending a brand to an identified group. For instance, they may believe that brand recommendations that take place in a close in-group relationship provide an opportunity for them to gain trust, recognition, and reputation (Koopman et al., 2015). A promotion-focused individual can make use of these advantages to achieve and maintain status within the collective with which they identify.

Moreover, identification with SBP users enhances conversation between users, which supports social connections within a group and helps to form a strong norm related to reciprocity within the collective (Wann, 2006). In this context, individuals with close categorical relationships are more likely to recommend brands because they trust the others in those relationships will return the favor and provide them with related information regarding other brands’ products. This provides an opportunity for promotion-focused individuals to expand new and useful resources, thereby satisfying their needs for advancement, growth, and accomplishment. Accordingly, identification with SBP users (vs. brand identification) is likely to lead to regulatory fit related to promotion and greater intentions to recommend brands among promotion-focused individuals. We therefore propose the following hypothesis.

**Hypothesis 6a:** *Identification with SBP users has more of an impact on brand recommendation than on brand identification for promotion-focused individuals.*

#### The Effects of Prevention Regulatory Fit on Brand Recommendations

Prevention-focused individuals are likely to be attuned to negative consequences in order to navigate mistakes and failure, and to ultimately reduce the chance of suffering losses (Higgins, 1997). Within an SBP, prevention-focused individuals may fear that the brands they recommend will be rejected, or will be unfairly criticized (Arazy and Gellatly, 2012). Brand identification means the brand’s identity is similar to the consumer’s identity (King et al., 2016). Identity similarly indicates that a brand or its products can convey attributes of the consumer’s self-definition, which help them to reduce uncertainty related to their identities (Wolter and Cronin, 2016). As such, this can effectively reduce prevention-focused individuals’ social anxiety when they recommend a brand with an identity that is congruent with their self-concept.

More importantly, brand identification suggests that a consumer perceives the brand’s identity as being prestigious or highly reputable (Bhattacharya and Sen, 2003). This attractive identity is positively associated with a brand’s ability to perform as expected and ensure product quality (He et al., 2012b; Ko et al., 2017). That is, brands with which individuals identify tend to be associated with higher levels of consumer trust, which can help prevention-focused individuals to avoid public disapproval and mitigate the risk associated with brand recommendations. In short, since brand identification helps to maintain the regulatory goal of prevention focus (preventing risks and avoiding negative outcomes), prevention fit will be achieved. Accordingly, the following hypothesis is proposed.

**Hypothesis 6b:** *Brand identification has more of an impact on brand recommendation than it does on identification with SBP users for individuals who are prevention focused.*

### The Moderating Effects of Product Type

#### Search Products and Regulatory Fit Effects

As previously explained, it is likely that consumers who are prevention oriented will take fewer risks and are more sensitive to uncertainty when they buy products (Higgins, 1998). Consumers can evaluate and compare search products’ main features prior to buying them, due to specific product descriptions (Wang et al., 2019). Available and concrete information regarding search products leads to lower levels of perceived uncertainty and a small psychological distance. As such, the search attributes of a target brand’s products can help prevention-focused individuals reduce risk, inducing greater repurchase intention. Additionally, when consumers have search products recommended to them, they tend to hold positive perceptions of them because recommended content regarding these products tends to be objective and so consumers can ascertain how reliable it is (Hsu et al., 2017). Taken together, lower levels of risk and uncertainty regarding search products are congruent with the characteristics of a prevention-focused mindset, resulting in greater intentions to repurchase and recommend search products. This eliminates the relative advantage of brand identification or identification with SBP users, resulting in no significant difference in the effects of the two identification targets for prevention-focused individuals.

However, individuals who are promotion focused tend to pay attention to ways to maximize potential gains; they employ risk-taking strategies (Higgins, 1998). That is, they pay less attention to lower levels of risk regarding search products, which does not hamper the original effectiveness of the two identification targets in brand repurchase and recommendations for promotion-focused individuals. Thus, the effects proposed in H5a and H6a hold for search products. Accordingly, we hypothesize the following.

**Hypothesis 7a:** *When a brand’s products belong to the search product category, brand identification (vs. identification with SBP users) will remain more effective in terms of brand repurchase intention for promotion-focused individuals. Conversely, two targets of identification will be comparably effective for prevention-focused individuals.*

**Hypothesis 7b:** *When a brand’s products belong to the search product category, identification with SBP users (vs. brand identification) will remain more effective in terms of brand recommendation for promotion-focused individuals. For prevent-focused individuals, targets of identification are likely to be relevant.*

#### Experience Products and Regulatory Fit Effects

Experience products’ attributes have to be assessed by consumers during or after consumption (Wang et al., 2019). This may better meet the exploration needs of promotion-focused individuals, due to the attention they pay to potential opportunities (Higgins, 1998). Following this logic, the experience attributes of a brand’s products tend to arouse promotion-focused consumers’ interest, which in turn may trigger greater intention to repurchase the brand’s products. Meanwhile, since the quality of experience-based products is difficult to observe before purchasing them, customers tend to look for other consumers’ evaluations of the products before making purchase decisions (Hsu et al., 2017). In this context, recommendations about experience products can help others to reduce the uncertainty of purchase, which provides an opportunity for promotion-focused individuals to gain higher status, boosting their self-image. Taken together, the attributes of experience-based products align with a promotion-focused mindset, which in turn enhances motivation to repurchase or recommend experience products, regardless of whether consumers identify with the brand or other SBP users. As such, the experience attributes of brand products hamper the effectiveness of two identification targets in regard to individuals who are promotion focused.

In contrast, prevent-focused individuals are more risk-averse (Higgins, 1998) and so a higher level of risk in regard to experience products reduces their repurchasing motivation. Furthermore, prevention-oriented individuals are likely to pay attention to others’ expectations and be afraid of public disapproval (Naletelich et al., 2019). Consequently, they may fear that recommendations regarding experience products will not be accepted, or will be questioned or even used against them due to more subjective evaluations in recommended content. All in all, the experience attributes of a brand’s products are inconsistent with the views of prevention-focused individuals and so do not affect the impact of social identification for these individuals, as postulated in H5b and H6b. Accordingly, we hypothesize the following.

**Hypothesis 8a:** *When a brand’s products belong to the experience product category, identification with SBP users (vs. brand identification) will remain more effective in terms of brand repurchase intention for prevention-focused individuals. Conversely, two targets of identification are effective for individual with a promotion focus.*

**Hypothesis 8b:** *If a brand’s products belong to the experience product category, brand identification (vs. identification with SBP users) will remain more effective in terms of brand recommendation for prevention-focused individuals. Conversely, two targets of identification will be comparably effective for promotion-focused individuals.*

**FIGURE 1:**
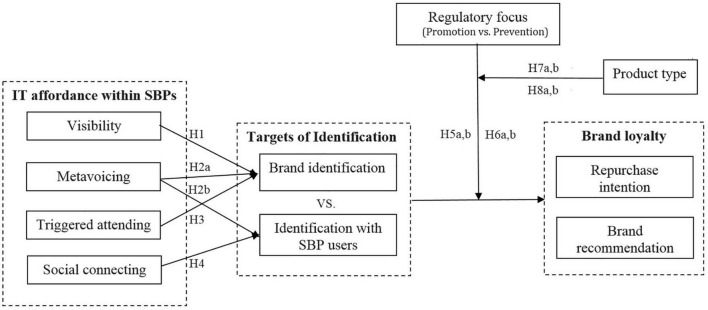
Research model.

With these hypotheses in mind, we create the research model portrayed in Figure 1. Overall, we seek to explore how IT affordances affect the formation of multiple identifications in regard to SBPs. Moreover, we investigate the relative effects of promotion fit and prevention fit on the different dimensions of brand loyalty when two types of individuals identify with the brand or other users of the SBPs. On this basis, we further examine whether the relative effects of promotion/prevention regulatory fit depend on the product type (search products vs. experience products). Finally, following previous literature (He et al., 2012b; Coelho et al., 2018; Santos et al., 2022), we test control variables such as age, gender, frequency of use of brand pages, and brand preference.

## Research Methodology

A mixed qualitative–quantitative research design is utilized. Specifically, we first use qualitative interviews to extract the relevant IT affordances that influence different impacts on the two targets of identification. Then, a quantitative survey is conducted to collect data and test the proposed model. The following sub-sections describe the method used in each process and the key measures.

### Interviews

Interviews were conducted with 27 participants who use social media. The participants met the following criteria: (1) at least 1 year worth of experience with SBPs; (2) members of at least two SBPs; and (3) used SBPs on a weekly basis. The sample included 12 men and 15 women; 14 of the respondents were in their 20 s, seven were in their 30 s, four were in their 40 s, and two were in their 50 s. A total of 10 respondents were students, five were freelancers/homemakers, and 12 were company workers. The profiles correspond closely to the demographic characteristics of SBP users (Chong et al., 2018). For example, the majority of SBP users are aged between 21 and 30 years, and student groups are an important part of SBPs. Previous studies have also shown that SBP users in China and the United States have the same demographic characteristics (Thongpapanl et al., 2018). Thus, the results suggest that our sample is fairly representative of SBP users in China and the United States. Prior to the interviews commencing, eligible respondents gave information about the SBPs they would refer to in the interviews, which enabled us to gain an understanding of how different SBPs operated. To thank them for participating in the research, each respondent was given a gift card.

We checked that each interviewee’s fan page was a brand fan page. Then, we asked what the interviewees could do through using SBPs (i.e., action possibilities). We then proceeded to ask deeper questions to unearth the effects of the affordances involved. We prompted interviewees by asking, for example, “How have action possibilities provided by the SBP influenced different targets of identification?” The respondents wrote down their answers on paper provided by the interviewer. Then, two coders carried out content analysis to identify which affordance was the root cause of the two identification targets. After the analysis of the interviews had been performed, we converted the results into percentages to represent the responses concerning different affordances (Table 1). Any category that exceeded the 20% cut-off point was marked as an antecedent of an identification target (Pavlou and Fygenson, 2006). Through this process, we identified the impacts of different affordances on the identification targets.

**TABLE 1:** The two identification targets within SBPs and their affordances.

Targets of identification	Visibility	Metavoicing	Triggered attending	Social connecting
1. Identification with SBP users	16.7%	**30.5%**	11.8%	**90.8%**
2. Brand identification	**48.2%**	**56.1%**	**55.9%**	16.2%

*Categories exceeding the 20% cut-off point are shown in bold and are underlined in the table.*

### Hypotheses Testing and Data Collection

To empirically examine the research model, especially the regulatory focus effect, we employed a survey company to recruit SBP users from the United States and China. We chose these two countries as research samples for the following reasons. First, because social media are employed extensively by users around the world (Hoehle et al., 2015), it is common for companies to run SBPs for followers with varied cultural backgrounds. Therefore, cross-cultural studies can assess whether patterns observed in one country can be generalized to another country. Second, previous studies suggest that cultural context influences individuals’ self-regulatory focus (Lee et al., 2000). Individualistic cultures tend to be promotion focused, aiming to achieve positive effects. In contrast, collectivist cultures tend to be prevention focused, aiming to avoid negative effects. According to Hofstede (2001) cultural indices, the United States and China are collectivist and individualist countries, respectively. In line with past research (Lockwood, 2005; Ashraf et al., 2017; Thongpapanl et al., 2018), we therefore recruited users of individual brand pages from the United States to represent promotion-focused Western culture and from China to represent prevention-focused Eastern culture.

The survey involved a screening process to ensure the responses would be of a high quality. Respondents were asked: (1) Do you follow a brand page on social media? (2) What is the name of the brand you follow most frequently? (3) Have you purchased and recommended this brand within the past 6 months? If they responded successfully to these screening questions, the participants could continue to complete the survey itself. Our study uses cross-sectional data mainly because it is commonly used in the area of human-computer interaction (Chong et al., 2018; Lazaroiu et al., 2020; Liu et al., 2021), making the results comparable and generalizable. Thus, the conclusions of this study are more convincing in the contribution they make to the field, due to the use of data of the same nature. We recruited 398 completed surveys from the United States and 416 from China. Table 2 shows the sample demographics.

**TABLE 2:** Sample demographic information.

Characteristics	Items	The United States (promotion-focused individuals)	China (prevention-focused individuals)
Gender	Male	38.8%	42.4%
	Female	61.2%	57.6%
Age	Below 18	3.7%	5.5%
	18–25	36.3%	40.8%
	26–35	22.5%	31.6%
	36–45	16.2%	15.7%
	46–55	8.8%	5.2%
	56 or over	12.5%	1.5%
Educational level	Less than high school	8.5%	6.3%
	High school graduate	26.5%	37.6%
	Bachelor’s degree	45.4%	38.7%
	Graduate degree	19.6%	17.4%
Usage frequency of brand pages	Less than once a week	15.2%	18.4%
	Once a week	11.1%	13.1%
	Several (<7) times a week	19.6%	13.8%
	Once a day	21.6%	19.5%
	Several times a day	32.5%	35.2%

### Measures

The constructs were measured with instruments that had been validated in previous research, with a few small adaptations made to the wording to ensure the constructs worked within the present research (please see Supplementary Appendix A). The construct instruments were rated on a seven-point Likert scale (“1 = strongly disagree” to “7 = strongly agree”). For use in China, the items were translated into Chinese. A standard back-translation procedure was carried out to make sure the Chinese version and the English version were consistent.

Prior to the survey process commencing, both versions were distributed to researchers, doctoral students, and SBP users, as a validity check, to refine the wording of the survey questions, to make sure the survey was easy to understand, and to specify any areas that needed improving before the survey went live. The majority (58.2%) of the participants in our sample are women and most (68.5) were aged between 18 and 45 years at the time. Most of the participants (69.7%) had used SBPs for more than 3 years and 35.2% of participants used their brand pages several times a day. The questionnaires were generally considered to be concise and accessible. The reviewers made some suggestions about the format and wording of some of the survey questions. Their suggestions were taken on board when we revised the survey. Afterward, the survey was pre-tested with a convenience sample of 132 respondents from both countries. The pretest yielded satisfactory reliability and validity measures.

### Data Analysis Method

We examined the possibility of common method bias arising because of the way the variables were all collected through a single survey; we used a pooled sample to do so, starting with a Harman one-factor analysis (Podsakoff et al., 2003). The majority (24.32%) of the variance could be explained by one factor. We then ran a model including a common method factor using SmartPLS (Liang et al., 2007). The results showed that 0.702 of the variance, on average, could be accounted for by the principle constructs. Using the averaged variance accounted for by the method factor resulted in a figure of 0.006. There was a ratio of 117:1 for the substantive variance to method variance. Only a few method factor loadings were significant and so it was deemed unlikely that common method bias was a serious issue in this study.

The current research used SmartPLS 3.0 to run a partial least squares (PLS) test in order to examine the psychometric properties of the scales and the research hypotheses. Supplementary Appendix A lists the constructs and their corresponding items used in the research method. We adopted PLS to conduct model validation for three reasons. First, PLS is a form of component-based structural equation modeling commonly used in exploratory research, such as in our study. Second, PLS does not massively restrict normal distributions (Leguina, 2015). Third, PLS works well with our model, which involves complex relationships, because it does not involve factor indeterminacy and inadmissible solutions in this context (Kim and Benbasat, 2006).

## Analysis and Results

### Measurement Model

Prior to running hypothesis tests, we checked the reliability, convergent validity, and discriminant validity of the measurement model. As shown in Table 3, each item was significant when loaded onto its respective construct and all of the loadings were above 0.50 (Hulland, 1999). The Cronbach’s alpha and composite reliabilities (CRs) were above 0.70. The average variance extracted (AVE) was greater than 0.50 (Table 4). The model can therefore be said to be reliable, with convergent validity. The model also has discriminant validity; the correlations between constructs were below 0.85 (Brown, 2006). The square root of the AVE for each construct was also above all of the correlations between each factor and the other constructs (Table 5). Therefore, the measures have satisfactory psychometric properties.

**TABLE 3:** Measurement model factor loadings.

Constructs	Items	Loading (the United States)	Loading (China)
Visibility	VI1	0.853	0.805
	VI2	0.775	0.859
	VI3	0.823	0.879
Metavoicing	ME1	0.887	0.823
	ME2	0.836	0.812
	ME3	0.811	0.765
Triggered attending	TA1	0.835	0.724
	TA2	0.841	0.903
	TA3	0.826	0.834
Social connecting	SC1	0.759	0.855
	SC2	0.829	0.848
	SC3	0.853	0.835
Brand identification	BI1	0.813	0.883
	BI2	0.823	0.918
	BI3	0.881	0.792
Identification with SBP users	UI1	0.848	0.778
	UI2	0.796	0.805
	UI3	0.779	0.866
Repurchase intention	RI1	0.926	0.919
	RI2	0.884	0.881
	RI3	0.802	0.825
Brand recommendation	BR1	0.835	0.771
	BR2	0.759	0.793
	BR3	0.733	0.835
Brand preference	BP1	0.824	0.817
	BP2	0.864	0.783
	BP3	0.757	0.818

**TABLE 4:** Cronbach’s α, CR, and AVE values for the constructs.

Construct	The United States (promotion-focused individuals)			China (prevention-focused individuals)		
	Cronbach’s α	CR	AVE	Cronbach’s α	CR	AVE
Visibility	0.718	0.855	0.686	0.856	0.832	0.759
Metavoicing	0.812	0.823	0.653	0.843	0.854	0.773
Triggered attending	0.833	0.849	0.762	0.823	0.923	0.743
Social connecting	0.785	0.874	0.673	0.867	0.976	0.765
Brand identification	0.822	0.812	0.689	0.828	0.854	0.623
Identification with SBP users	0.912	0.864	0.776	0.876	0.983	0.754
Brand repurchase	0.806	0.851	0.743	0.842	0.856	0.637
Brand recommendation	0.792	0.864	0.642	0.756	0.823	0.664
Brand preference	0.833	0.856	0.778	0.885	0.839	0.768

**TABLE 5:** Correlations between the constructs and the square roots of the AVEs (on the diagonal).

The United States (promotion-focused individuals)	1	2	3	4	5	6	7	8	9
1. Visibility	**0.828**								
2. Metavoicing	0.132	**0.808**							
3. Triggered attending	0.255	0.254	**0.873**						
4. Social connecting	0.218	0.134	0.141	**0.820**					
5. Brand identification	0.236	0.231	0.327	0.160	**0.830**				
6. Identification with SBP users	0.216	0.361	0.265	0.174	0.307	**0.881**			
7. Brand repurchase	0.277	0.217	0.243	0.183	0.137	0.163	**0.906**		
8. Brand recommendation	0.122	0.346	0.223	0.145	0.316	0.425	0.263	**0.801**	
9. Brand preference	0.215	0.329	0.167	0.177	0.328	0.237	0.365	0.145	**0.882**

**China (prevention-focused individuals)**	**1**	**2**	**3**	**4**	**5**	**6**	**7**	**8**	**9**

1. Visibility	**0.871**								
2. Metavoicing	0.142	**0.879**							
3. Triggered attending	0.265	0.254	**0.862**						
4. Social connecting	0.047	0.177	0.264	**0.875**					
5. Brand identification	0.139	0.127	0.053	0.166	**0.789**				
6. Identification with SBP users	0.171	0.154	0.159	0.179	0.235	**0.868**			
7. Brand repurchase	0.365	0.353	0.143	0.272	0.266	0.184	**0.798**		
8. Brand recommendation	0.233	0.272	0.163	0.091	0.186	0.378	0.322	**0.815**	
9. Brand preference	0.258	0.148	0.075	0.181	0.168	0.483	0.354	0.252	**0.876**

*The diagonal elements (in bold) are the square root of AVEs, and off-diagonal elements are correlations.*

### Structural Model

We first estimated the direct impacts of IT affordances on two targets of identification (Table 6) with subsamples of the United States (highly promotion oriented) and China (highly prevention oriented) participants. No significant differences were found in age, gender, or education in the subsamples, following a one-way ANOVA. Specifically, in all of the subsamples, visibility exerts positive effects on brand identification, supporting H1. Metavoicing exerts positive effects on two targets of identification, supporting H2a and H2b. Social connections exert positive effects on identification with SBP users, supporting H4. In addition, triggered attending had a significant positive impact on brand identification in the China subsample, supporting H3.

**TABLE 6:** Values from model testing on the basis of nations.

Hypothesis	Promotion-focused individuals (the United States)	Prevention-focused individuals (China)
H1: Visibility → brand identification	0.266***	0.272***
H2a: Metavoicing → brand identification	0.374***	0.363***
H2b: Metavoicing → identification with SBP users	0.252***	0.228***
H3: Triggered attending → brand identification	0.075	0.136**
H4: Social connecting → identification with SBP users	0.121*	0.129**
***Control variable***		
Gender → repurchase intention	0.02	0.137**
Gender → brand recommendation	0.06	0.09
Age → repurchase intention	−0.156**	−0.08
Age → brand recommendation	−0.04	−0.123*
Usage frequency of brand pages → repurchase intention	−1.74***	−0.05
Usage frequency of brand pages → brand recommendation	−0.02	−0.07
Brand preference → repurchase intention	0.243***	0.265***
Brand preference → brand recommendation	0.378***	0.315***

*p < 0.05; **p < 0.01; ***p < 0.001.

To explore the regulatory focus effects, we compared the path coefficients from two identification targets to different dimensions of brand loyalty in relation to the United States and China. Specifically, we use the following formula from Yang et al. (2020):

***T***_(*path coefficient difference*)_ = (path coefficient_1_ – path coefficient_2_)/SQR[(SE_1_^2^ + SE_2_^2^)/N]

Where N represents the sample size and SE represents the standard error.

As shown in Table 7, brand identification has more of an impact than identification with SBP users does in relation to repurchase intention for promotion-focused individuals, supporting H5a. However, for prevention-focused individuals, identification with SBP users has more of an impact than brand identification does on repurchase intention, validating H5b. Our results also confirm H6a, indicating that identification with SBP users has a stronger impact than brand identification does on brand recommendation for promotion-focused individuals. Furthermore, prevention-focused individuals engender stronger positive brand recommendations when they identify with the brand in question, providing support for H6b.

**TABLE 7:** Analytic results of the regulatory fit effects.

Path coefficient		Path coefficient difference	Supported
**Promotion-focused individuals (the United States)**			
H5a	β_BI→RI_ vs. β_UI →RI_ = 0.532*** vs. 0.321***	β = 0.211***	Supported
H6a	β_UI→ BR_ vs. β_BI→ BR_ = 0.336*** vs. 0.178***	β = 0.158***	Supported
**Prevention-focused individuals (China)**			
H5b	β_UI→RI_ vs. β_BI →RI_ = 0.378*** vs. 0.215***	β = 0.163***	Supported
H6b	β_BI→BR_ vs. β_UI →BR_ = 0.477*** vs. 0.258***	β = 0.219***	Supported

BI, brand identification; UI, identification with SBP users; RI, repurchase intention; BR, brand recommendation. ***p < 0.001.

### The Moderating Effects of Product Type

To analyze the hypothesized moderating effects, we tested whether the two conditions (promotion fit vs. prevention fit) engender different effects on brand loyalty based on product category. Accordingly, we coded the sample manually, with each brand selected by the participants categorized as a “search” (Group 1) or an “experience” (Group 2) brand. To ensure the sorting process was reliable, two coders from a relevant research field who were not otherwise associated with the current study were invited to assign the self-selected brands to the previously defined categories. We did not inform them of our hypotheses in order to prevent our expectations from affecting their coding. After they completed the independent coding, the two coders discussed any discrepancies to reach mutual agreements. Pairwise agreement rates were 89% and 92% for Group 1 and Group 2, respectively, indicating satisfactory reliability (Rust and Cooil, 1994).

Using the aforementioned formula form Yang et al. (2020), a multi-group analysis was conducted in order to ascertain whether the two group path coefficients were different in any significant way. As shown in Table 8, promotion-focused individuals who identify with a brand are more likely to repurchase search products. However, the effects of the two identification targets on repurchase intention in regard to search products do not significantly differ for prevention-focused individuals. Hence, these results support H7a. Likewise, promotion-focused individuals who identify with SBP users are more willing to recommend search products. Conversely, the effect of the two identification targets on recommendations of search products do not significantly differ for prevention-focused individuals. These results confirm H7b.

**TABLE 8:** Analytic results of the moderating effects of product type.

Product type		Path coefficient	Path coefficient difference	Supported
***Search products***				
H7a	Promotion-focused individuals (the United States)	β_BI→*RI*_ vs. β_UI →*RI*_ = 0.355*** vs. 0.173***	β = 0.182***	Supported
	Prevention-focused individuals (China)	β_UI→*RI*_ vs. β_BI→*RI*_ = 0.278*** vs. 0.258***	β = 0.020 (n.s.)	
H7b	Promotion-focused individuals (the United States)	β_UI→BR_ vs. β_BI→BR_ = 0.463*** vs. 0.256***	β = 0.207***	Supported
	Prevention-focused individuals (China)	β_BI→BR_ vs. β_UI→BR_ = 0.256*** vs. 0.237***	β = 0.019 (n.s.)	
***Experience products***				
H8a	Prevention-focused individuals (China)	β_UI→RI_ vs. β_BI→RI_ = 0.302*** vs. 0.261***	β = 0.041 (n.s.)	Partially
	Promotion-focused individuals (the United States)	β_BI→RI_ vs. β_UI→RI_ = 0.282*** vs. 0.217***	β = 0.065 (n.s.)	Supported
H8b	Prevention-focused individuals (China)	β_BI→BR_ vs. β_UI→BR_ = 0.432*** vs. 0.238***	β = 0.194***	Supported
	Promotion-focused individuals (the United States)	β_UI→BR_ vs. β_BI→BR_ = 0.204*** vs. 0.171***	β = −0.033 (n.s.)	

*BI, brand identification; UI, identification with SBP users; RI, repurchase intention; BR, brand recommendation.*

***p< 0.001; and n.s., non-significant.

On the other hand, for experience products, prevention-focused individuals have significantly stronger recommendation intentions when they identify with a brand. Conversely, two targets of identification have the same effect for promotion-focused individuals. Thus, H8b is supported. Yet, similar to promotion-focused individuals, we did not find a significant difference between the two identification targets in regard to repurchase intention for experience products for prevention-focused individuals. Thus, H8a is only partially supported. We interpret these findings in the section “Discussion.”

### *Post hoc* Analysis

In order to further confirm the robustness of promotion and prevention focus classified by country, participants were invited to respond to the regulatory focus scale to distinguish promotion/prevention focus from their personal perspectives. Specifically, following the work of Higgins (1998), we calculated the differences between the averages of the promotion and prevention measures. We then categorized the respondents as being either promotion focused or prevention focused, based on the median split in the different measures. Finally, we had 403 promotion-focused individuals and 386 prevention-focused individuals. Table 9 shows that the relative impacts of promotion/prevention regulatory fit on the two dimensions of brand loyalty remain the same, offering further evidence that our results are robust.

**TABLE 9:** *Post hoc* analysis of regulatory fit effects.

Path coefficient		Path coefficient difference	Supported
**Promotion-focused individuals**			
H5a	β_BI→RI_ vs. β_UI →RI_ = 0.505*** vs. 0.318***	β = 0.187***	Supported
H6a	β_UI→ BR_ vs. β_BI→ BR_ = 0.341*** vs. 0.208***	β = 0.133***	Supported
**Prevention-focused individuals**			
H5b	β_UI→RI_ vs. β_BI →RI_ = 0.356*** vs. 0.207***	β = 0.149***	Supported
H6b	β_BI→BR_ vs. β_UI →BR_ = 0.434*** vs. 0.221***	β = 0.213***	Supported

*BI, brand identification; UI, identification with SBP users; RI, repurchase intention; BR, brand recommendation.*

***p < 0.001.

### Discussion

We discuss three interesting results of this study. As predicted, the results confirm that the relative impacts of the two identification targets on brand loyalty are associated with different regulatory focuses (H5a, H5b, H6a, and H6b). This finding is in contrast to previous research, which focuses only on either brand identification or identification with SBP users (Warner-Soderholm et al., 2018; Kaur et al., 2020; Martínez-López et al., 2021). Our research shows that, when consumers with different regulatory focuses face two identification targets (i.e., the brand and SBP users), there may be promotion or prevention regulatory fit, which can enhance repurchase or recommendation intentions. *Post hoc* analysis confirms that the effect of regulatory fit on brand loyalty is consistent, regardless of whether regulatory focus is classified by individual or by country. This contributes to previous research that has called for the role of regulatory fit to be compared at different levels (Chang et al., 2019).

Second, our findings show how product type can moderate the influence of promotion/prevention regulatory fit on two dimensions of brand loyalty. Even if previous studies suggest that an individual’s regulatory fit positively impacts brand loyalty (Wu and Hsu, 2015), research has not explored boundary conditions, and so the context in which regulatory fit occurs has not been investigated. On the one hand, our study confirms that the search attributes of products can significantly change the relative importance of the two identification targets to brand loyalty (H7a, H7b). On the other hand, in terms of experience products, our study also proves that prevention fit consumers recommend brands to a greater extent, and the two identification targets have the same effect for promotion-focused individuals (H8b).

Yet, unlike H8a, identification with SBP users is no more effective than brand identification in increasing repurchase intention for prevention-focused individuals. One possible explanation for this finding is that the SBP takes place on a third-party platform (social media, for example), which has institutional mechanisms in place to provide protection for consumers. Previous research has shown that if the institutional mechanism on a platform is stronger, consumers will be less concerned about risks related to sellers’ actions (Chong et al., 2018). For prevention-focused customers, institutional mechanisms thus decrease perceptions of uncertainty related to experience products under the SBP environment, which in turn contributes to their repurchase intentions. Consequently, this eliminates the advantage of identification with SBP users among prevention-focused customers, causing the two identification targets to be comparably effective in increasing repurchase intention.

Third, with affordances in mind, we conducted an initial qualitative study. In doing so, we identified four antecedents of multiple forms of identification in terms of SBP. These four antecedents are: visibility, metavoicing, triggered attendance, and social connections. Many prior studies have shown that empirical explorations of IT affordances in the SBP context are still in their infancy (Lin et al., 2019). To fill this gap, our empirical results confirm that nearly all IT affordances examined in this study significantly predicted different identification targets (H1, H2a, H2b, H3, H4). Put another way, testing the model within two different cultures, the United States and China, allowed us to extend the results and show the validity of the model, explaining the role of IT affordances in building a strong level of identification with a brand itself and other users of the SBP. Our findings also contribute to a deeper understanding of IT applications in social contexts, in relation to the links between individuals and technology (Piccoli, 2016).

## Conclusion

### Implications for Theory

First, we extend the extant literature on brand loyalty in the SBP context by unveiling the different roles that two identification targets play in increasing customer loyalty. Since brand loyalty includes two dimensions with different attributes, such as brand repurchase and brand recommendation, they have different connections with the antecedents at hand. This theoretically illustrates that only considering one identification target is inadequate. To overcome these shortcomings, our research represents a preliminary effort to simultaneously integrate brand identification and identification with other SBP users in a dual identification framework. Specifically, we deepen and expand our knowledge of the relative importance of different identification targets in relation to the two dimensions of brand loyalty within SBPs.

Second, the present research is the first to extend the applicability of regulatory focus theory to the context of SBPs, which have thus far remained largely unexamined. Specifically, prior research has found a positive effect of social identification on brand loyalty within SBPs, but has not examined how this effect is different for different individuals with distinct regulatory focuses (e.g., individuals who are promotion focused vs. individuals who are prevention focused). This research therefore fills the gap by combining regulatory focus theory and social identification theory, and ascertains that the fit concerning two identification targets and the regulatory focus of customers can impact brand loyalty in significant ways. More importantly, given that individuals’ regulatory orientations vary between Eastern and Western cultures, we investigate the aforementioned effects of promotion/prevention regulatory fit on brand loyalty across the United States and China.

Third, this research enhances our understanding of the moderating role of product type (search vs. experience products) on brand loyalty in the SBP context. We examine how interactions between product type and regulatory fit affect brand loyalty in terms of repurchasing and brand recommendations. Our results show how the relative importance of promotion and prevention regulatory fit for brand loyalty depends on product type. Thus, the current research has taken a first step toward confirming the moderating effects of product type on the connection between promotion/prevention regulatory fit and brand loyalty within SBPs.

Fourth, the present research expands the applicability of theory concerning IT affordances, which act as a vital foundation for IS research. Using both qualitative interviews and quantitative surveys enabled us to examine precise combinations of IT affordances. Our results also indicate that many IT affordances support both “social” and “commercial” elements for SBP users, and thus play important roles in enabling consumers’ brand loyalty. As such, the present research emphasizes the theoretical and managerial importance of IT affordances, as well as expanding the theoretical applications involved in order to generate a deeper and broader understanding of IT affordances in the context of SBPs.

### Practical Implications

This study’s analysis and results have practical implications. First, the results of the research suggest that two identification targets have different impacts on brand loyalty between promotion-focused and prevention-focused individuals. Accordingly, individuals with different regulatory focuses should first be identified by companies, which would then help companies to find incentives that fit their customers. Then, companies should further design and use aligned incentive strategies that are congruent with either a promotion-focused or prevention-focused mindset. Specifically, in order to satisfy individuals’ identification with SBP users, companies could offer users chances to participate in activities with other users. For individuals who fit the brand identification, companies should help them to address any issues with complementary products or services, and resolve users’ complaints in an efficient way.

Second, the current research confirms that product type moderates the impact of regulatory fit on brand loyalty. Thus, companies should investigate where brand success originates (i.e., within the brand itself or through other SBP users) and adopt strategies in relation to the identification targets, depending on product type. For search products, companies should focus on promoting adequate identification targets to strengthen the effect of prevention regulatory fit on two dimensions of brand loyalty. Conversely, for experience products, companies strive to implement a series of effective social identification strategies to increase the role of promotion regulatory fit on brand loyalty. In a nutshell, companies selling different product types need to identify suitable targets and work to understand ways to work with them in order to build brand loyalty in relation to SBPs.

Finally, users’ social identification is affected by the four IT affordances under study in this research. Therefore, companies can use this research to inform the ways they use SBPs. For example, companies could make use of the IT affordances embedded in their platforms to increase the power of SBPs’ social and commercial impact. Furthermore, companies could use IT affordances to inform their design of other features to develop the range of affordances they have on offer. Specifically, they could consider how to improve interpersonal interactions and ease the minds of users with precise location-sensitive and other customized information, knowing that these elements do indeed affect social identification within SBPs.

### Limitations and Future Research

This study also has some limitations. First, the conceptual model concerns consumers’ identification with both brands and other SBP users as key determinants of brand loyalty. Other factors may also have important effects; therefore, future research could incorporate additional factors into the research model (e.g., individuals’ online behavior, prior purchasing experience, and social influence) in order to enrich our understanding of consumer loyalty within SBPs.

Second, to investigate the moderating effects of product type, we classified product types into search products and experience products, following previous research (Peterson et al., 1997). The extent to which the findings of this study can be generalized to other contexts is thus limited to these items. Future research could explore a variety of ways of classifying products, to ascertain whether the findings differ in different contexts.

Third, we tested the hypotheses using cross-sectional data, and so it was only possible to determine causal relationships between independent and dependent variables. However, the nature of social identification and brand loyalty may change over time. To develop existing knowledge regarding these variables, future longitudinal studies could be conducted. Longitudinal data may also reduce common method bias (Podsakoff and Organ, 1986).

## Data Availability Statement

The raw data supporting the conclusions of this article will be made available by the authors, without undue reservation.

## Author Contributions

SC and QM were carried out and determined the formal analysis and methodology. SC was carried out the writing of the original draft and reviews of the writing and editing. XX was conducted the data curation. All authors have finalized and are in agreement with the published version of the manuscript.

## Conflict of Interest

The authors declare that the research was conducted in the absence of any commercial or financial relationships that could be construed as a potential conflict of interest.

## Publisher’s Note

All claims expressed in this article are solely those of the authors and do not necessarily represent those of their affiliated organizations, or those of the publisher, the editors and the reviewers. Any product that may be evaluated in this article, or claim that may be made by its manufacturer, is not guaranteed or endorsed by the publisher.
