# Supplementary material for: Understanding the Relative Impact of Dual Identification on Brand Loyalty on Social Media: The Regulatory Fit Perspective in Different Cultures
Source: Front Psychol. 2022 Jun 14;13:901706. doi: 10.3389/fpsyg.2022.901706 (PMC9237455; doi:10.3389/fpsyg.2022.901706)
Supplement: Supplementary file 1 [file Table_1.pdf]

Appendix A. Constructs and item descriptions

Constructs	Items	Sources
Visibility (VI)	**VI1:** Using the brand's social media enables me to access information about products or services I intend to use or need.<br>**VI2:** The brand's social media gives me access to detailed photographs of products.<br>**VI3:** The brand's social media makes the product attributes visible to me.	(Rice et al., 2017)
Metavoicing (ME)	**ME1:** The brand's social media allows me to comment on products.<br>**ME2:** The brand's social media allows me to react to companies' feedback on products.<br>**ME3:** The brand's social media enables me to provide others with information about my shopping experiences.	(Majchrzak et al., 2013)
Triggered attending (TA)	**TA1:** The brand's social media gives me information about product changes.<br>**TA2:** The brand's social media promotes product information.<br>**TA3:** The brand's social media gives me information about product upgrades.	(Dong et al., 2016)
Social connecting (SC)	**SC1:** The brand's social media gives me access to information about products because of the connections I am able to form with other consumers.<br>**SC2:** The brand's social media lets me make connections with others and we are able to talk about products together.<br>**SC3:** The brand's social media connects me with others who can provide information about products.	(O'Riordan et al., 2016)
Brand identification (BI)	**BI1:** The brand is important to me.<br>**BI2:** The brand believes in the same things as I do.	(Stokburger-Sauer et al., 2012)

	**BI3:** The way I perceive the brand and the way I perceive myself are similar.	
Identification with SBP users (UI)	**UI1:** I feel connected to other people on the brand's social media.<br>**UI2:** Other people on the brand's social media share objectives with me.<br>**UI3:** I am a user of the brand's social media.	(Algesheimer et al., 2005)
Repurchase intention (RI)	**RI1:** I am likely to purchase the brand's products in the future.<br>**RI2:** I will continue to buy the brand's products.<br>**RI3:** If I were to buy the same product again, the brand would be my first choice.	(Jang et al., 2008)
Brand recommendation (BR)	**BR1:** I will leave positive comments about this new brand on SBPs.<br>**BR2:** I am likely to recommend the brand.<br>**BR3:** I often talk to others about the brand.	(Chaudhuri and Holbrook, 2001)
Brand preference (BP)	**BP1:** Although there are other brands providing similar products, I would rather purchase products from this brand.<br>**BP2:** Even if other brands are the same as this brand, I still prefer to buy from this brand.<br>**BP3:** Although other brands provide products with the same features as this brand does, I would still rather buy products from this brand.	(Kim and Hyun, 2011)

REFERENCES

Algesheimer, R., Dholakia, U. M., and Herrmann, A. (2005). The social influence of brand community: Evidence from European car clubs. J. Marketing Res. 69, 19-34. doi: 10.1509/jmkg.69.3.19.66363

Chaudhuri, A., and Holbrook, M. B. (2001). The Chain of Effects from Brand Trust and Brand Affect to Brand Performance: The Role of Brand Loyalty. J. Marketing 65, 81–93. doi: 10.1509/jmkg.65.2.81.18255

Dong, X., Wang, T., and Benbasat, I. (2016). "IT affordances in online social commerce: conceptualization validation and scale development," in Proceedings of Twenty-Second Americas Conference on Information Systems, (San Diego).

Jang, H., Olfman, L., Ko, I., Koh, J., and Kim, K. (2008). The influence of online brand community characteristics on community commitment and brand loyalty. Int. J. Elect. Commerce 12, 57–80. doi: 10.2753/JEC1086-4415120304

Kim, J.-H., and Hyun, Y. J. (2011). A model to investigate the influence of marketing-mix efforts and corporate image on brand equity in the IT software sector. Industrial Marketing Manage. 40, 424–438.doi: 10.1016/j.indmarman.2010.06.024

Majchrzak, A., Faraj, S., Kane, G. C. and Azad, B. (2013). The Contradictory Influence of Social Media Affordances on Online Communal Knowledge Sharing. J.Comput.Mediated Commun. 19 (1), 38-55.doi: 10.1111/jcc4.12030

O'Riordan, S., Feller, J., and Nagle, T. (2016). A categorisation framework for a feature-level analysis of social network sites. J. Decision Syst. 25, 244–262. doi: 10.1080/12460125.2016.1187548

Rice, R. E., Evans, S. K., Pearce, K. E., Sivunen, A., Vitak, J., and Treem, J. W. (2017). Organizational Media Affordances: Operationalization and Associations with Media Use. J. Commun. 67, 106–130. doi: 10.1111/jcom.12273

Stokburger-Sauer, N., Ratneshwar, S., and Sen, S. (2012). Drivers of consumer–brand identification. Int. J. Res. Marketing 29, 406–418. doi: 10.1016/j.ijresmar.2012.06.001
